# Sulfur Speciation in Li–S Batteries Determined by *Operando* Laboratory X-ray Emission Spectroscopy

**DOI:** 10.1021/acsaem.4c02330

**Published:** 2024-11-20

**Authors:** Ava Rajh, Alen Vizintin, Joanna Hoszowska, Robert Dominko, Matjaž Kavčič

**Affiliations:** † Jožef Stefan Institute, Jamova 39, 1000 Ljubljana, Slovenia; ‡ Faculty of Mathematics and Physics, University of Ljubljana, Jadranska Ulica 19, 1000 Ljubljana, Slovenia; ¶ National Institute of Chemistry, Hajdrihova 19, 1000 Ljubljana, Slovenia; § Physics Department, University of Fribourg, Chemin du Musée 3, CH-1700 Fribourg, Switzerland

**Keywords:** lithium−sulfur batteries, X-ray emission spectroscopy, *operando* measurements, oxidation state, von Hamos spectrometer

## Abstract

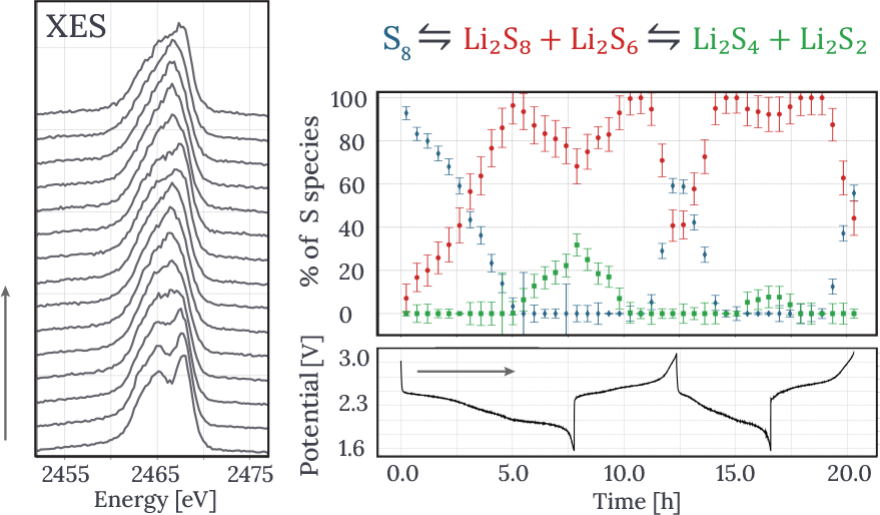

In this work, *operando* sulfur X-ray emission measurements on a Li–S battery cathode were performed using a laboratory setup as an alternative to more common synchrotron radiation based absorption studies. Photoexcitation by an X-ray tube was used. Valence-to-core Kβ X-ray emission spectra were recorded with a wavelength dispersive crystal spectrometer in von Hamos geometry, providing excellent energy resolution and good detection efficiency. The setup was used to record *ex situ* S Kβ emission spectra from S cathodes from the Li–S battery and also under *operando* conditions. Average S oxidation state within the battery cathode during battery cycling was determined from the shape of the Kβ emission spectra. A more detailed S species characterization was performed by fitting a linear combination of previously measured laboratory synthesized standards to the measured spectra. Relative amounts of different S species in the cathode were determined during the cycling of the Li–S battery. The main advantage of X-ray emission spectroscopy is that it can be performed on concentrated samples with S loading comparable to a real battery. The approach shows great promise for routine laboratory analysis of electrochemical processes in Li–S batteries and other sulfur-based systems under *operando* conditions.

## 1 Introduction

Recent advancements in synchrotron radiation instrumentation over the past decades, coupled with the extensive theoretical work used to interpret the measured data, established synchrotron radiation based X-ray spectroscopy as one of the most powerful tools for researchers in all fields of natural sciences, including solid state physics, catalysis, energy applications, and environmental sciences.^1^ However, limited and competitive access to large-scale synchrotron facilities has recently increased the interest in performing X-ray experiments with in-house laboratory sources.^2,3^ The most conventional X-ray technique employed in material structure analysis is X-ray absorption spectroscopy (XAS). Taking benefit from experimental concepts developed at synchrotron beamlines, XAS has been successfully adapted for laboratory use by using X-ray tubes with various anode materials. However, most of the currently available modern laboratory XAS setups^4−9^ are restricted to the hard X-ray energy range above 5 keV and operate in transmission mode. Such an experimental approach is not applicable for the analysis of bulk materials composed of light elements (Al, S, Cl), with absorption edges situated in slightly lower tender energy range ∼1.5–5 keV, which typically requires in-vacuum measurements in fluorescence mode. In addition, XAS measurements are limited to diluted samples due to the self-absorption effect, which do not represent a realistic battery.

Local electronic structure of bulk materials can also be studied with high energy resolution X-ray emission spectroscopy (XES). Unlike XAS, XES does not require monochromatic tunable photon beam, making it particularly suitable for laboratory analysis. Emission spectra are independent of the excitation mode, and different laboratory sources of ionizing radiation can be used to produce the initial core–hole state. While XAS is often the preferred method for laboratory studies in the hard X-ray range, with first successful applications in the field of energy and catalysis recently reported,^10,11^ XES is a favorable option for laboratory electronic structure analysis in the tender X-ray range.^12−14^

One particularly promising application of laboratory-based X-ray spectroscopy is the characterization of next-generation batteries. With their high energy density, low-cost, and environmentally friendly S cathode, lithium–sulfur (Li–S) batteries are a leading candidate for the next-generation of energy-storage technologies.^15−17^ The ability to perform routine *operando* laboratory structural and chemical analysis of electrochemically active materials would represent an important step toward speeding up further technological development. In our recent work,^18,19^ laboratory S XES has been used to characterize electrochemical S conversion within Li–S batteries. The emission spectra of S are also not affected by target self-absorption, and the measurements can be performed on concentrated samples, much more similar to a realistic working battery. While our previous results demonstrated high sensitivity of XES to S electronic structure and even feasibility for quantitative analysis of the electrochemical S conversion in a Li–S battery, they have been restricted to *ex situ* measurements on precycled cathodes. The use of a high-energy incident proton beam caused radiation damage, making it challenging to maintain proper electrochemistry while keeping the beam parameters (proton energy and beam current) sufficiently high to obtain good-quality XES spectra throughout the battery cycle.

In this work, further advancements toward full *operando* analysis have been made by replacing MeV proton excitation with more traditional photoexcitation using an X-ray tube. In addition, a von Hamos XES spectrometer^20^ equipped with the cylindrically curved highly annealed pyrolytic graphite (HAPG) analyzer crystal has been used to enhance X-ray detection efficiency. This experimental setup has been used to first record the S Kβ XES spectra from a series of precycled cathodes extracted from Li–S batteries stopped at different points. Finally *operando* measurements of Kβ spectra were performed on a Li–S battery during two consecutive discharge/charge cycles. Electrochemical conversion of S during cycling was tracked by the shape of the Kβ emission spectra, and detailed S species characterization was performed with a linear combination fit of laboratory synthesized standards.

## 2 Experimental Section

### 2.1 Materials Preparation

The S cathode composite was prepared using Ensaco 350G (Imerys) carbon, which was ball milled with S for 30 min at 300 rpm in a mass ratio of 1:2. The mixture was then heated in a quartz tube under argon at a rate of 0.2 °C min^–1^ to 155 °C, held at that temperature for 5 h, and subsequently cooled to room temperature. The S content was around 66 wt %

#### 2.1.1 Preparation of *Ex Situ* Electrodes

Electrodes for the ex situ measurements were prepared by mixing the carbon/sulfur composite (66 wt % S), polyvinylidene fluoride (PVdF) binder, and conductive multiwalled carbon nanotubes (NTL, M-grade) in a mass ratio of 8:1:1. The slurry was prepared in *N*-methyl-2-pyrrolidine (NMP) and cast on a carbon-coated aluminum foil. The typical S loading on carbon-coated aluminum foil was approximately 2.3 mg of S cm^–2^. A pouch-type two-electrode cell was prepared inside an argon filled glovebox. The sulfur cathode (2 cm^2^ electrode) was separated from the metallic lithium anode with glass fiber separators (GF-A from Whatman). The electrolyte, consisting of 1 M LiTDI (Solvionic, 99.9%) in tetraethylene glycol dimethyl ether (TEGDME):1,3-dioxolane (DOL) (1:1 vol %), was used in excess (60 μL per mg of sulfur). The cells were cycled and stopped at different potential values, such as the middle of the first plateau (2.4 V), the end of the high voltage plateau (2.39 V), the middle of the sloping region (2.18 V), the beginning of the second plateau (2.0 V), the middle of the second plateau (2.04 V), the end of the second plateau (1.97 V), and the full discharge at 1.5 V by using a Maccor 4200 galvanostat/potentiostat at a current density of C/10 (167.2 mA g^–1^). All cells were disassembled inside an argon-filled glovebox and vacuum sealed in a pouch bag with a 3.6 μm thick Maylar window.

#### 2.1.2 *Operando* Cell Assembly and Electrochemistry

Electrodes were prepared by mixing the carbon/sulfur composite (66 wt % sulfur), polytetrafluoroethylene (PTFE) binder, and conductive multiwalled carbon nanotubes (NTL, M-grade) in 2-propanol, in a mass ratio of 8:1:1. The self-standing electrodes (1.13 cm^2^) were pressed onto a carbon-coated aluminum mesh collector (3.14 cm^2^) and dried overnight at 50 °C. The sulfur loading on the self-standing electrode was 3.4 mg. The two electrode cell for operando measurement was assembled in an argon filled MBraun glovebox using an custom-made *operando* vacuum-tight Swagelok cell with a 6 μm thick Mylar foil plated with 500 Å of aluminum on the side facing the cathode.^21^ The sulfur cathode was separated from the metallic lithium anode by two glass fiber separators (GF-A from Whatman). The electrolyte, consisting of 1 M LiTDI (Solvionic, 99.9%) in tetraethylene glycol dimethyl ether (TEGDME):1,3-dioxolane (DOL) (1:1 vol %), was used in excess (100 μL per mg of sulfur). Due to the harsh conditions, including the battery being in a high vacuum and exposed to the X-ray beam, a high amount of electrolyte was used to ensure good electrochemical performance and to prevent the electrolyte quantity from limiting the performance. The battery was cycled between 1.5 and 3.0 V using a Bio-Logic SP-200 galvanostat/potentiostat at a current density of C/30 (55.7 mA g^–1^).

### 2.2 XES Measurements

XES measurements were conducted at the University of Fribourg, Switzerland. X-ray emission from the samples was induced by irradiation with bremsstrahlung from a Coolidge-type side-window Sc X-ray tube with a Be window of 0.15 mm, operating at 40 kV and 35 mA. XES spectra were recorded with a high-resolution in-vacuum spectrometer in von Hamos geometry.^20,22,23^ A cylindrically bent HAPG(001) (2*d* = 6.708 Å) crystal with 25.4 cm curvature radius focused the reflected X-rays onto a back-illuminated charged-coupled device (CCD) detector (1340 × 400 pixels, with pixel dimensions of 20 μm × 20 μm) thermoelectrically cooled to −45 °C. A 0.2 mm wide rectangular slit defined the source size. Samples were positioned behind the slit, where the center of the slit and the center of the crystal were aligned along the direction determined by the central Bragg angle. The fwhm energy resolution at the energy of the S KB transition was 0.8 eV. The detector positions were converted to an energy scale using the Kα_1_ and Kβ_1,3_ peaks of elemental S_8_ with corresponding reference energies of 2307.89 eV ^24^ and 2467.96 eV,^25^ respectively.

Initially, *ex situ* Kβ XES spectra were obtained from eight precycle sulfur cathodes in Li–S cells with the cells halted at different discharge points. The acquisition times for each cathode ranged from 15 to 30 min. The samples were mounted on a rotating stage and encased in vacuum-sealed pouches prepared in Ar atmosphere. *Operando* XES measurements were performed on a vacuum-tight Swagelok cell, mounted within the vacuum chamber. Spectra were recorded from the back of the battery cathode. Two consecutive discharge–charge cycles were monitored. Spectra were recorded every 10 min, and 3 such measurements were summed together for the final acquisition times of around 30 min. A linear background was subtracted from the final spectra and each spectrum was normalized to the area under the Kβ peak.

## 3 Results and Discussion

### 3.1 *Ex Situ* XES on Precycled Sulfur Cathodes

**Figure 1:**
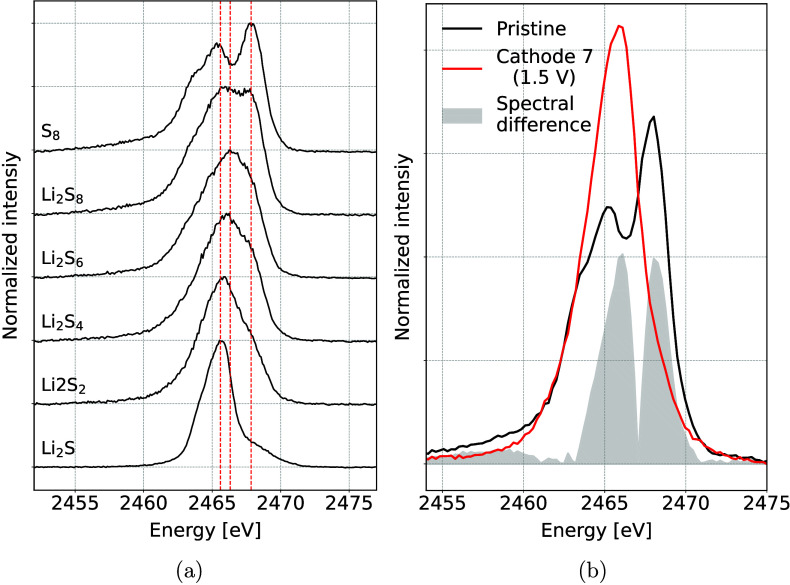
(a) Kβ emission spectra of polysulfide standards recorded in our previous work.^18^ (b) Spectra of pristine cathode and a precycled cathode at the end of battery discharge. The spectral difference used to determine the IAD value is shown in gray. The three vertical lines show the positions of three energy peaks belonging to Li_2_S, Li_2_S_6_, and S_8_, respectively.

S Kβ spectra correspond to transitions from occupied valence states, and the spectral shape reflects occupied valence molecular orbitals. They contain detailed information about local electronic structure around S atoms, their bonding and symmetry. In our previous work,^18^ a series of Kβ XES spectra on reference compounds (S_8_, Li_2_S_*x*_; *x* = 8...1) were recorded and can be seen on Figure 1a. The polysulfide standards were synthesized by mixing stoichiometric amounts of lithium metal scraps and sulfur. By extracting the integrated absolute difference (IAD) between the pure elemental S_8_ reference and the measured Kβ spectra of polysulfides, a quantification parameter was established. The IAD parameter was shown to have approximately linear correlation with the average S oxidation state in a sample, which can be used to determine the S oxidation state in each individual battery cathode.^18^ Graphical representation of the IAD parameter is shown in Figure 1b. The same approach was applied in our current work.

**Figure 2:**
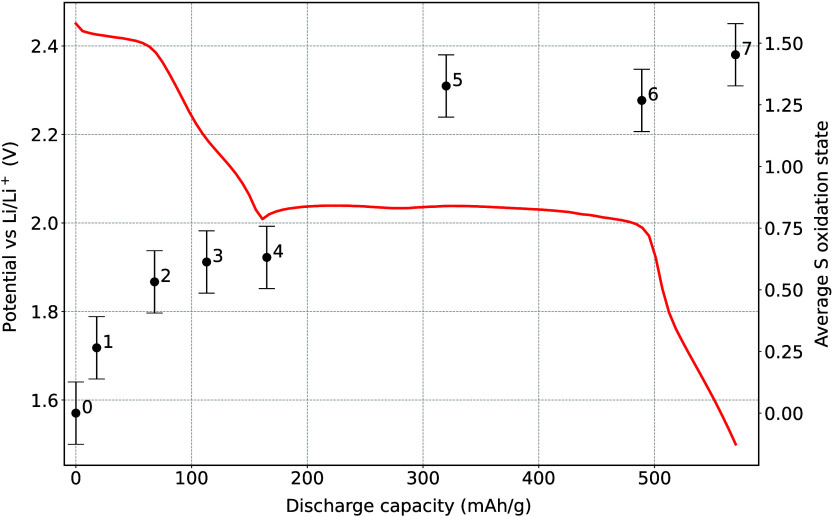
Average S oxidation state in *ex situ* battery cathodes, determined from the differences of the Kβ emission spectra. Red line shows galvanostatic discharge curve.

The IAD values were determined for spectra of seven precycled cathodes stopped at different points during battery discharge and a spectrum collected from pristine cathode. The values were converted to the average S oxidation state in samples using a previously established linear relation. The final oxidation states are shown in Figure 2 along with a discharge curve of a typical Li–S cell from which the electrodes were taken. It shows a gradual increase in the average S oxidation state with the battery discharge. The change in the first part of the cycle is faster, indicating a quick reduction of elemental S_8_ into long chain polysulfides during the first plateau and a more gradual change in oxidation state in the latter part of the low voltage plateau, where most of the long chain polysulfides have already reduced. In this latter part of the electrochemistry curve, a transition from soluble short-chained polysulfides into crystalline Li_2_S occurs. The maximum S oxidation state does not reach the theoretically predicted value of −2 for Li_2_S, implying an incomplete reduction of S. This result is consistent with both XES and XAS measurements performed at synchrotron facilities and with our previous *ex situ* measurements using a proton beam as X-ray excitation source.^18,26,27^

**Figure 3:**
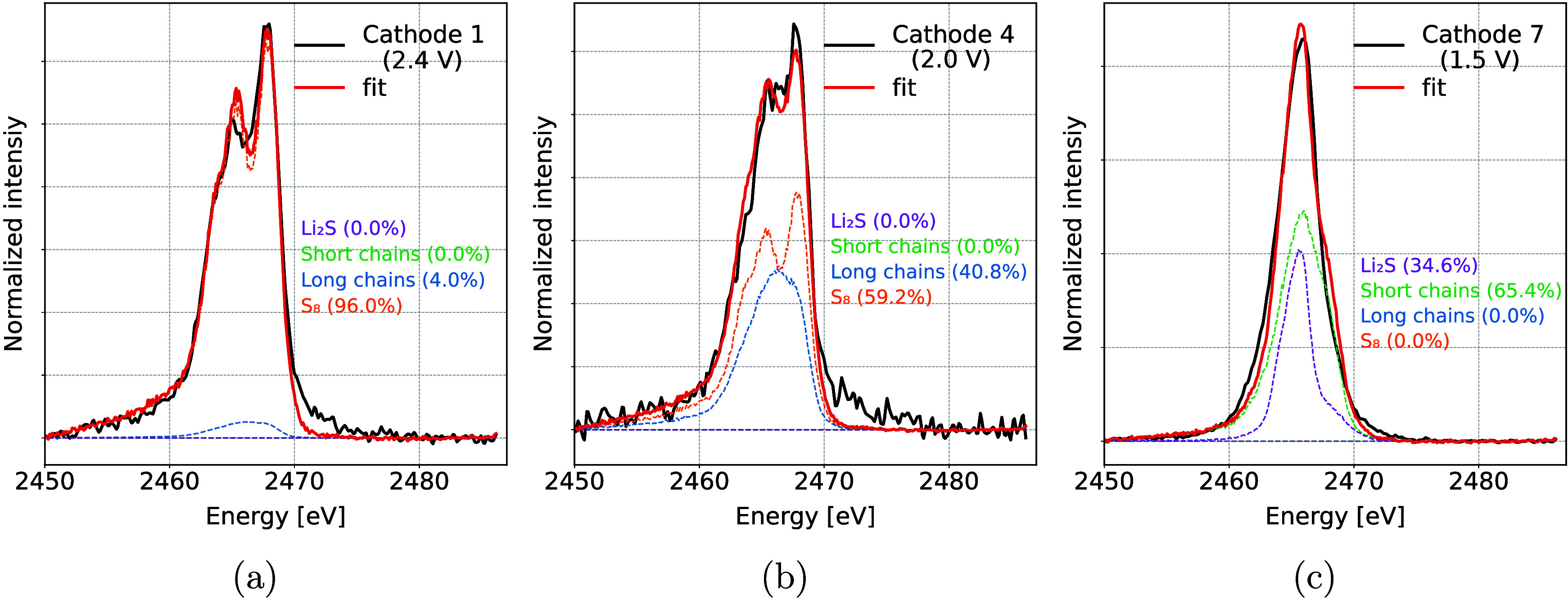
Kβ emission spectra of three *ex situ* battery cathodes and the best fit with a linear combination of reference standards at (a) beginning of the high voltage plateau, (b) beginning of the low voltage plateau, and (c) end of the discharge.

**Figure 4:**
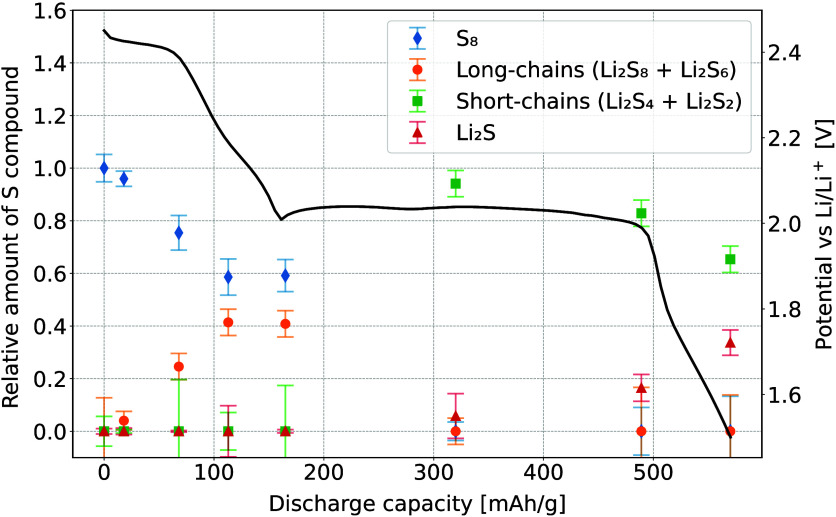
Relative intensities of S components within the battery samples during the discharge. Black line shows the galvanostatic discharge curve.

To separate the contribution from different polysulfide species in the samples, a linear combination fit (LCF) with the spectra of Li_2_S_*x*_ standards was performed. Since the laboratory-synthesized standards usually contain a mixture of Li_2_S_*x*_ polysulfides with a mean S chain length corresponding to *x*, the spectra of reference standards Li_2_S_8_ and Li_2_S_6_ were added into a single long-chained polysulfide signal and Li_2_S_4_ and Li_2_S_2_ into a general signal of short-chained polysulfides. In addition, pure elemental S_8_ and Li_2_S were also included in the fit. The fit was performed using nonlinear least squared minimization using python lmfit package.^28^ First a model function was constructed as a weighted sum of three reference components. During optimization, the weights are adjusted to minimize the difference between the combined model and the measured data. Constraints placed on the weights ensured they were non-negative. Few examples of the fit can be seen in Figure 3, and the relative amounts of each component in the *ex situ* cathodes are presented in Figure 4 along with the typical discharge curve. During the first plateau, exhibiting a fast change in S oxidation states, the elemental S is being reduced into long-chained polysulfides. In the first half of low voltage plateau, these long-chains are then reduced into short-chained polysulfides, part of which are then gradually further reduced into crystalline Li_2_S. The final S composition at the end of the discharge consists of about 35% in the form of Li_2_S and 65% bound into short-chained polysulfides. This is not surprising as mixed short-chain polysulfides/Li_2_S structures are present due to disproportionation reaction and not full electroreduction.^29^

Formation of crystalline Li_2_S during the low-voltage plateau was also observed in previous *operando* XAS and RIXS measurements performed at synchrotrons.^21,26,27^ The latter shows Li_2_S becoming a major species by the end of the discharge. Less efficient conversion has been observed in this study, with Li_2_S reaching about one-third of the total sulfur amount. Considering the nominal oxidation state for the Li_2_S (−2) and the Li_2_S_2_/Li_2_S_4_ short-chained polysulfides (−0.75), this ratio can be converted to an average S oxidation state of approximately −1.2, which is slightly lower compared to the oxidation state obtained from the IAD value in Figure 2. This discrepancy is larger at the beginning of the discharge, where cathodes contain larger amounts of long-chained polysulfides. This is a result of systematic deviations of the IAD values measured for longer Li_2_S_*x*_ (*x* = 4, 6, 8) standards from the linear calibration curve used to convert the IAD values into the average S oxidation state.^18^

### 3.2 *Operando* XES on a Li–S Battery

**Figure 5:**
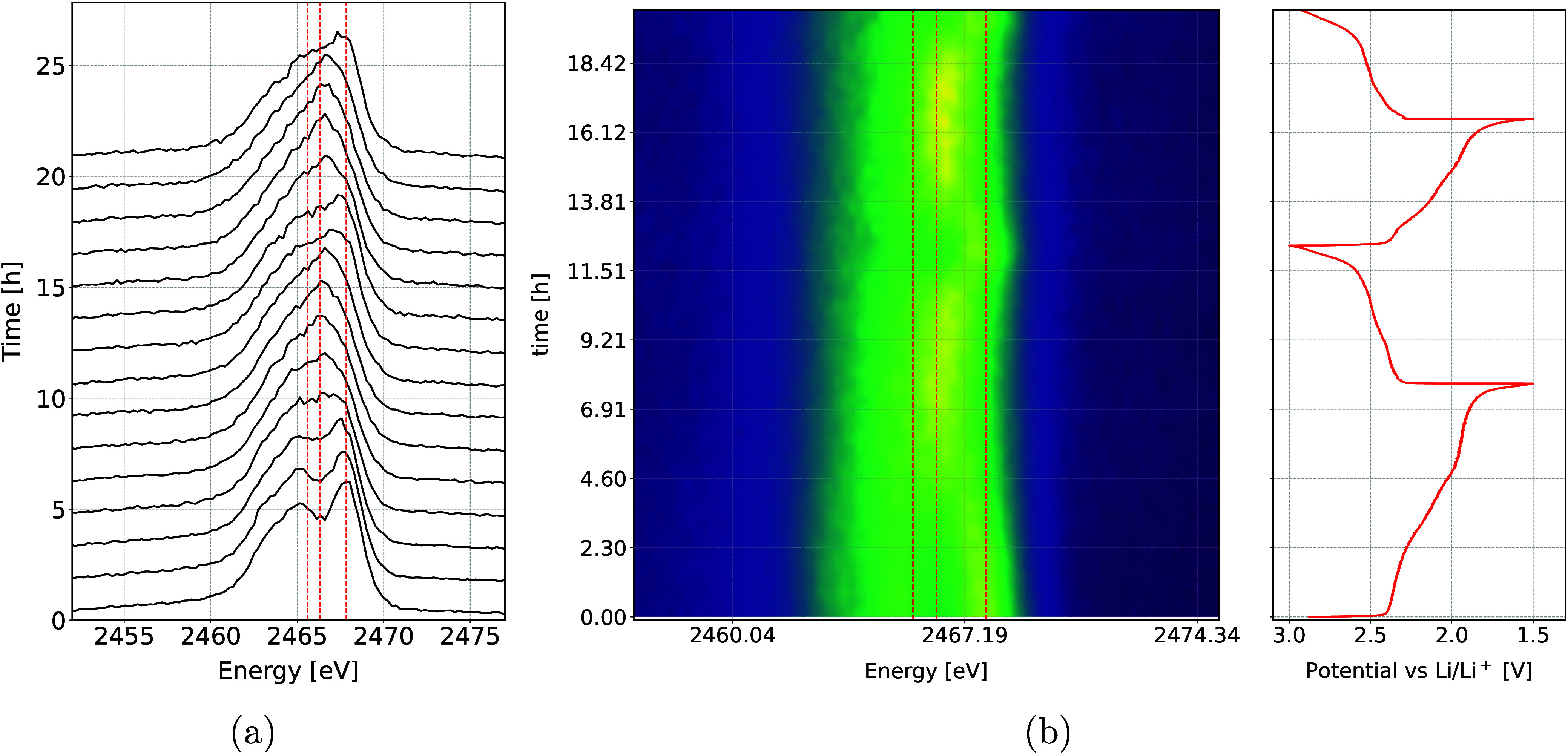
(a) Kβ S emission spectra recorded during battery cycling. (b) Heat map showing Kβ S emission spectra, normalized to the same area, during the battery cycling (left) along with the galvanostatic discharge/charge curve (right). The three vertical lines in (a) and (b) show the positions of three energy peaks belonging to Li_2_S, Li_2_S_6_, and S_8_, respectively.

The goal of this research was to perform a simultaneous laboratory XES analysis during the Li–S battery operation. *Operando* measurements represent the most genuine insight into the electrochemical processes within the battery. They avoid any inherent variations of the *ex situ* measurements resulting from different individual batteries cycled in parallel and any interference with the original battery assembly. *Operando* S Kβ emission spectra were recorded every 10 min during two discharge/charge cycles. Three consecutive spectra were added together for a cumulative time resolution of 30 min and normalized to the same area. The resulting series of the measured spectra during two full cycles is displayed in a 2D plot in Figure 5 along with the galvanostatic curve of the Li–S cell. A reduction of pure elemental S_8_ to polysulfides can be observed. The doublet structure of S_8_ becomes weaker as the transition into long-chain polysulfides occurs during the high voltage plateau and in the first sloping region. This is followed by a clear and rather sharp transition at the beginning of the low voltage plateau into a more prominent and symmetric peak, characteristic for short-chained polysulfides. During the subsequent charge, this transition reverses. In the beginning of the second discharge/charge cycle the doublet structure is less prominent and complete reduction of pure S happens faster, as there are some unreduced polysulfides left after the charge.

**Figure 6:**
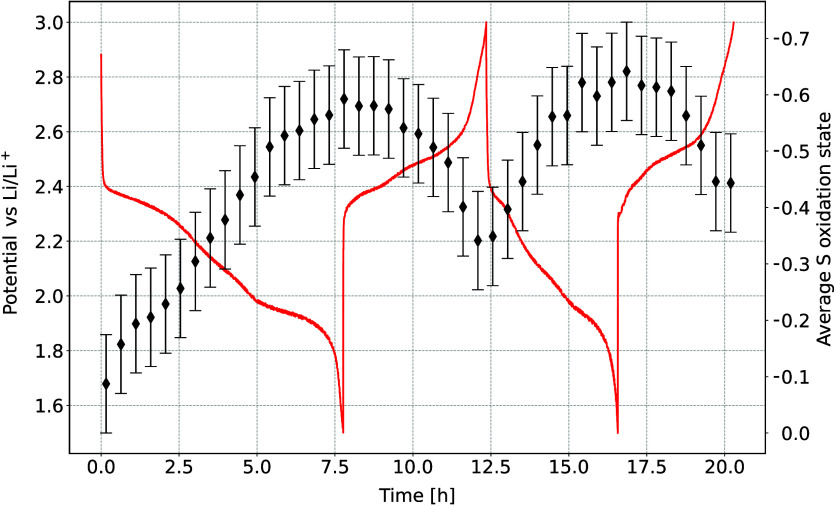
Average S oxidation state during battery cycling, determined from the IAD values between the consecutive *operando* Kβ emission spectra and the corresponding initial spectrum. Red line shows the galvanostatic discharge curve.

As for the *ex situ* spectra, the IAD relative to the spectra of the initial battery state was determined from the measured *operando* spectra and converted to the average S oxidation state. The evolution of the oxidation state can be seen in Figure 6 and shows a trend similar to that in the *ex situ* samples. The change in the oxidation state occurs rapidly and follows a linear relationship up to the beginning of the low voltage plateau. For the remainder of the discharge cycle, the changes are more gradual, reaching the ultimate average S oxidation state of −0.6 by the end of the discharge. During the following charge cycle, the average S oxidation state decreased to −0.35, indicating that some polysulfides remained dissolved in the electrolyte and did not oxidize back to pure S_8_. Despite the imperfect S conversion, the battery successfully underwent further cycling. In the following discharge, the first part of the electrochemistry curve was shortened as there was less S_8_ available. However, during the low voltage plateau, the same average S oxidation state was reached as in the first cycle. The further loss of capacity at the end of the second charge suggested that a larger portion of S dissolved in the electrolyte did not fully revert back to pure S_8_, confirming a gradual loss of active material over multiple discharge/charge cycles. Relatively low final oxidation state by the end of the discharges implies little or no amount of Li_2_S with a formal oxidation state of −2. This is in contrast to the results of *ex situ* measurements, where the final oxidation state by the end of the discharge was determined to be −1.5. Low content of Li_2_S at the end of the discharge cycle was attributed to incomplete electrochemical conversion, indicated also by the short low voltage plateau. This was attributed to radiation damage from the exposure to X-rays during cycling. The precycled battery cathodes were discharged without the presence of an X-ray beam, and the electrochemical reaction proceeded as expected.

**Figure 7:**
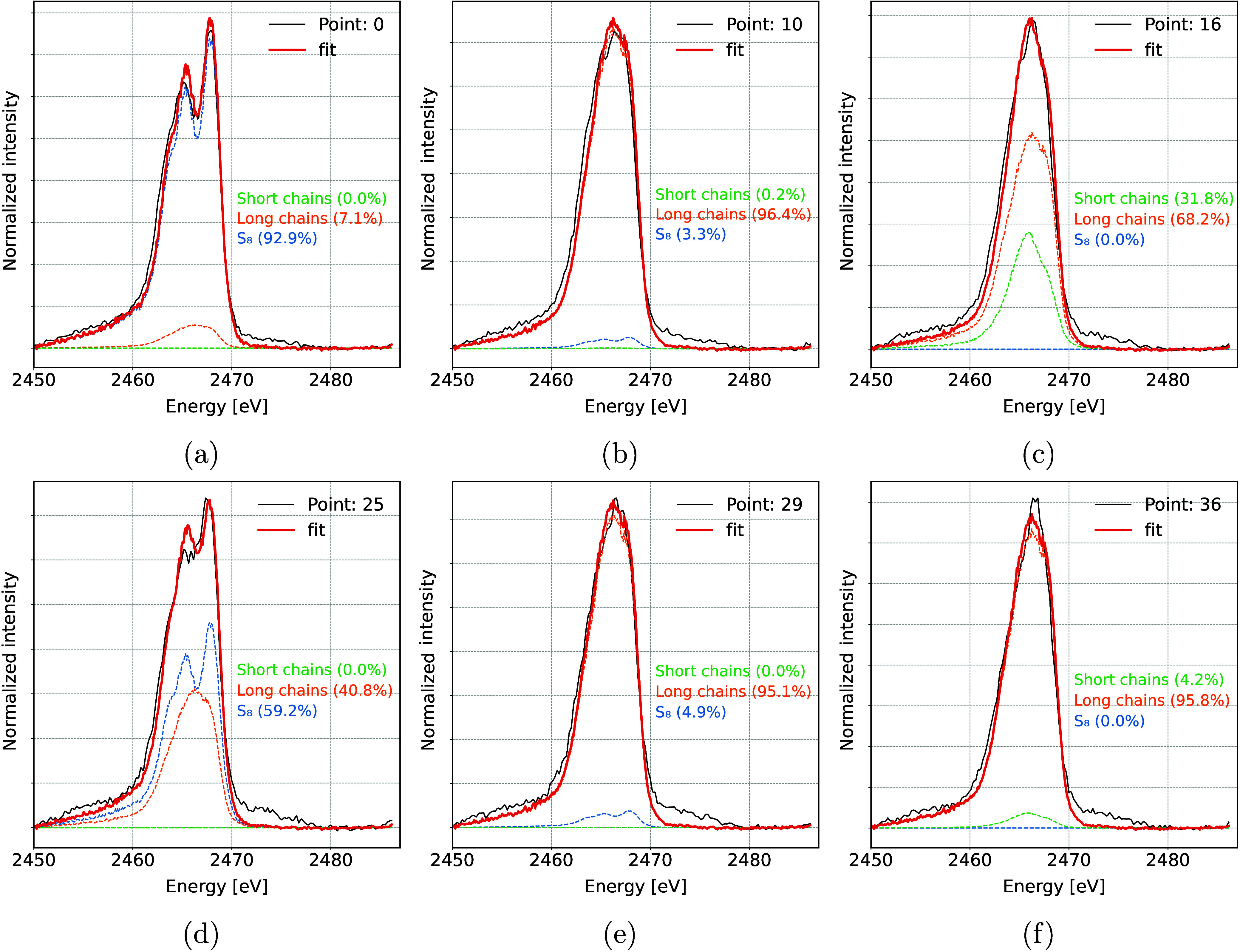
Examples of Kβ emission spectra recorded during *operando* measurements and the best fit with a linear combination of reference standards. The shown spectra correspond to (a) start of discharge at the beginning of the high voltage plateau, (b) beginning of the low voltage plateau, where all S is reduced into long-chained polysulfides, (c) end of first discharge, (d) beginning of second discharge, (e) start of the second low voltage plateau, and (f) end of second discharge.

A more detailed quantitative analysis of different sulfur species present in the battery during cycling was performed with an LCF of *operando* XES spectra. All measured spectra were described with a linear combination of three components: pure elemental S_8_, long-chained polysulfides (a normalized sum of Li_2_S_8_ and Li_2_S_6_ standards), and short-chained polysulfides (a normalized sum of Li_2_S_4_ and Li_2_S_2_ standards). No Li_2_S component was detected at the end of the discharge cycle; therefore, it was excluded from the analysis. This could be due to measurements from the back side of the cathode and small undetectable amounts of Li_2_S resulting from a shorter low-voltage plateau. Examples of the LCF fit on the *operando* spectra at different points during battery cycling are shown in Figure 7.

**Figure 8:**
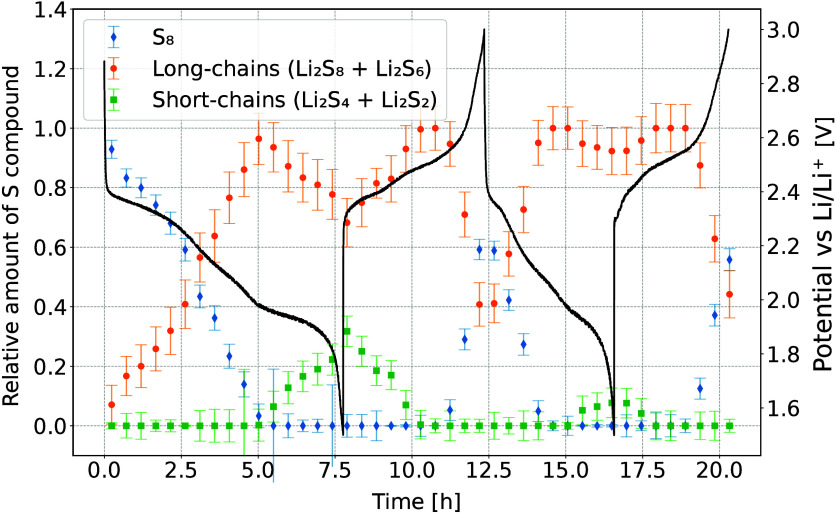
Relative intensities of different S species within the battery during two consecutive discharge/charge cycles. Black line shows the galvanostatic curve.

Relative amounts of each component determined from the fits are shown in Figure 8. In the initial state, only pure S_8_ is present in a battery. A fast reduction of S_8_ is seen in the beginning of discharge and during the high voltage plateau, accompanied by a simultaneous increase in the presence of long-chained polysulfides. By the start of the low voltage plateau almost all of the S_8_ is reduced, and the dominant process becomes conversion of longer polysulfides into shorter ones. Maximum amount of S bound in short-chained polysulfides is found at the end of the first discharge and corresponds to about 30% of all present S. At the beginning of the next charge cycle, the order of reactions reverses. The amount of elemental S returns to about 60% of the original amount, with 40% of it remaining bound in long-chained polysulfides. While the average S oxidation state in Figure 6 reaches the same value after the first and second discharges, the characterization of S species shows some difference between both cycles. In the first discharge, a combination of long- and short-chained polysulfides is present by the end of discharge, while the amount of short-chained polysulfides is much lower at the end of second discharge.

Comparing the galvanostatic curve of the Li–S cell during *operando* measurements with a typical curve for the precycled cathodes measured *ex situ*, it is evident that the low voltage plateau in both *operando* cycles is much shorter. This is reflected in the S oxidation state at the end of the discharge, which matches the oxidation state at the beginning of the low voltage plateau measured *ex situ* for precycled cathodes, and the absence of any Li_2_S in *operando* spectra at the end of both cycles. This was attributed to the radiation damage induced by the X-ray exposure of the battery. It is known that the latter can hinder the electrochemical reaction by causing restricted charge transfer, migration of Li ions, and damage to the electrolyte.^30^ Other factors, such as S loading, the amount of electrolyte, and sulfur/carbon ratio, all influence the cell electrochemistry. Introducing various catalysts in the carbon matrix for sulfur can also greatly affect the performance of the Li–S cell, due to faster conversion kinetics for short-chained polysulfides into Li_2_S.^31−33^ At this stage, we were primarily interested in the feasibility of the proposed experimental approach; therefore, the changes in the electrochemistry were not critical for the purpose of this work.

## 4 Conclusion

Laboratory XES spectroscopy was used to provide a detailed S speciation in the Li–S battery during cycling. The chemical sensitivity of S Kβ emission spectra was used to determine the average S oxidation state and quantitatively analyze relative amounts of different S species within the S cathode. The *ex situ* measurements on precycled samples are consistent with previously obtained results using both XAS and XES, showing a gradual decrease in average S oxidation state during battery discharge. LCF analysis also showed a reduction of elemental S_8_ until the start of the low voltage plateau where long-chained polysulfides transformed first into short-chained polysulfides which then partially reduced into Li_2_S by the end of the discharge. By performing XES *operando* measurements, the S oxidation state was tracked through two consecutive discharge/charge cycles. The results showed an incomplete S reduction during both discharges, reaching about −0.6 average S oxidation state. Detailed S species analysis via LCF fitting was able to track the reduction of pure S_8_ along with the formation of long-chained polysulfides in the high voltage plateau. In the low voltage plateau, most of the elemental S has already reduced and a conversion of long to short-chained polysulfides follows. No crystalline Li_2_S was detected at the end of discharge due to a short low voltage plateau.

Laboratory XES is thus shown to be an effective and accurate S speciation method for *operando* measurements on Li–S batteries. The measurements can be performed on concentrated samples without any dilution, which is typically required in XAS analysis to reduce self-absorption. They have excellent time resolution, and the results are comparable to XAS analysis performed at synchrotron facilities. The accessibility of this method allows for continuous monitoring of S species over dozens of cycles near the home laboratory without the constraints of limited synchrotron beam time allocation. In addition, quicker analysis times would mean faster iteration and would speed up development and synthesis of new materials for Li–S batteries. However, radiation damage observed in our study suggests the need for further enhancement of the overall experimental efficiency to reduce battery X-ray exposure. This study demonstrates the capability of laboratory-based XES spectroscopy for sulfur speciation in Li–S and other metal–S batteries, opening the door for more routine *operando* analysis on numerous battery samples over large number of cycles and development of a novel battery cell that would enable more efficient reduction/oxidation processes.
